# Estimating bilateral hand kinetics from upper-limb kinematics in sprint front crawl: a subject-wise validation of an artificial neural network

**DOI:** 10.3389/fbioe.2026.1978998

**Published:** 2026-10-15

**Authors:** Tianyi Gao, Shang Qi, Jiawen Li, Dongxiang Huang, Kaiqi Zheng, Haixia Li, Jie Gao

**Affiliations:** 1 School of Education, Beijing Sport University, Beijing, China; 2 School of Physical Education and Sports Science, South China Normal University, Guangzhou, China; 3 Yale College, Yale University, New Haven, CT, United States; 4 School of Physical Education, Shaoguan University, Shaoguan, China; 5 School of Physical Education, Shanxi University, Taiyuan, China; 6 Langfang Sports School, Langfang, Hebei, China

**Keywords:** artificial neural network, front crawl swimming, hand propulsive force, mechanical power, sports biomechanics, statistical parametric mapping, underwater motion capture

## Abstract

**Objective:**

Hand force contributes substantially to front crawl performance, but measuring it requires hand-worn hardware. We tested whether an artificial neural network driven only by three-dimensional upper-limb kinematics can reconstruct bilateral hand kinetics recorded by a hand-force device, and at which level of the signal (waveform, discrete descriptors, between-swimmer and inter-limb differences) the estimates agree.

**Methods:**

Twenty-four male competitive swimmers performed maximal 50-m front crawl without breathing while wearing eo SwimBETTER handsets; 14 upper-body markers were tracked by underwater motion capture at 100 Hz. For each swimmer, the underwater phase (fingertip entry to exit) of each hand was analysed. A laterally mirrored multilayer perceptron using 197 trunk-frame features estimated six targets per hand under subject-wise six-fold cross-validation, with architecture, regularisation and smoothing selected inside each fold. It was compared with four alternative learned models and two baselines (a scalar group-mean null model and a phase-conditioned mean-waveform template), and uncertainty was estimated with participant-cluster bootstraps.

**Results:**

For resultant force, root-mean-square error was 24.5 ± 6.1 N (left) and 23.3 ± 9.9 N (right), per-window correlations were 0.85 and 0.84, and ICC(A,1) was 0.70 and 0.64. The network was comparable to gradient boosting and coordinate-only models but was not more accurate than the template (template RMSE 21.8 and 21.2 N; left-hand difference 2.7 N, 95% CI 0.9–4.5). Between-swimmer correlations of measured and estimated peak force, impulse and mean force ranged from −0.36 to 0.27, and the estimated inter-limb symmetry index did not agree with the measured one (r −0.23 to −0.10; the dominant side was matched in 50%–58% of swimmers). Left-hand peak force was underestimated by 32.6 N, and the absolute error in time to peak averaged 12%–13% of the underwater phase. Modelling both hands in every window, a broader definition that includes each hand’s recovery and entry, inflated apparent accuracy (left RMSE 21.6 N, r 0.89).

**Conclusion:**

In a device-on protocol, kinematics-driven estimation recovered the group-typical force–time pattern but added little to a phase template and did not resolve differences between swimmers or between limbs. It cannot yet replace hand-force measurement for individual assessment.

## 1 Introduction

In front crawl, the force the hands apply to the water contributes substantially to performance, yet it cannot be measured with a standard laboratory instrument. There is no aquatic equivalent of the force platform: the athlete is unsupported and the reaction is taken up by a fluid that itself moves. Pressure-based approaches estimate hand force from the palmar–dorsal pressure differential and the hand’s surface area, combined either with underwater motion capture (Tsunokawa et al., 2019; Koga et al., 2022) or with a hand-mounted inertial measurement unit (Kadi et al., 2022), and the sensors themselves can disturb the flow they sample. Computational fluid dynamics avoids instrumentation but requires individualised hand geometry and substantial computation per condition (Bixler and Riewald, 2002; Marinho et al., 2011). Tethered and flume protocols are simpler but alter the task (Ruiz-Navarro et al., 2024; Sacilotto et al., 2023). Commercial hand-worn devices provide in-pool kinetic outputs with acceptable test–retest reliability (Santos et al., 2022; Bastián-Antón et al., 2025), but the hardware must be purchased and fitted, and it cannot be applied retrospectively. Wearable waterproof electromyography and inertial sensing are a lower-cost complement, although the signal integrity and reproducibility of such sensors in water must themselves be established before biomechanical interpretation (Amin et al., 2023; Amin et al.,2025). Propulsive kinetics therefore remain largely confined to specialised laboratories.

Kinematics are in a different position. Underwater motion capture and video are already used in many programmes, and markerless pose estimation is reducing the labour that once limited them (Giulietti et al., 2023). Kinematic archives therefore accumulate faster than synchronised force records. If hand force could be inferred from kinematics that are already collected, propulsion could be studied in more sessions, more athletes and historical datasets. On land, machine learning has shown potential for an analogous problem: convolutional networks estimate ground reaction forces from joint kinematics with normalised errors below 13% in healthy individuals and below 21% in cerebral palsy (Ozates et al., 2025), and embedding dynamics improves prediction (Kim et al., 2020). Whether comparable accuracy is attainable in water is not obvious. Ground reaction arises from contact tightly constrained by whole-body kinematics, whereas hand propulsion arises from unsteady hydrodynamics that depend on hand velocity, orientation, effective area and vortex dynamics (Takagi et al., 2021), only some of which can be recovered from marker coordinates. Kinematics therefore carry information about hand force but do not determine it.

A second question is how such an estimator should be judged. Many machine-learning studies in biomechanics report a single pooled R^2^ and partition records rather than subjects, both of which inflate apparent accuracy (Halilaj et al., 2018). For a cyclic waveform, a pooled R^2^ is dominated by within-cycle variation and can remain high even when the model discriminates poorly between individuals, which is precisely the ability a coach needs. Two further safeguards are needed. First, the benchmark set should include a model that knows only the typical shape of the force–time curve, so that credit is given only for information beyond that shape. Second, the analysis window should follow the accepted definition of the stroke phase, so that easy portions of the signal (for example, a hand out of the water) do not inflate agreement.

This study therefore aimed (1) to develop a subject-independent artificial neural network that estimates bilateral hand force components and device-derived power indices from three-dimensional upper-limb kinematics, and to benchmark it under subject-wise, nested cross-validation against learned alternatives and a phase-conditioned template; and (2) to evaluate its estimates at the waveform, discrete-variable, between-swimmer and inter-limb levels, so that the boundary of their applicability is established. The contribution is the bilateral, multi-target validation rather than a new network architecture. We hypothesised that the network would reproduce the force–time waveform more completely than it would represent between-swimmer magnitude differences.

## 2 Materials and methods

### 2.1 Participants

Twenty-four male competitive swimmers participated (Table 1). The sample size was set by the number of eligible national-level and regional-level male sprinters who could complete the full underwater motion-capture and hand-force protocol during the data-collection period, not by an *a priori* power calculation; it exceeds the ten swimmers of the device validation study (Bastián-Antón et al., 2025). All were free of upper-limb injury in the preceding 6 months and were competing at national or regional level, with a mean 50-m freestyle season best of 24.01 ± 0.60 s (745 ± 35 World Aquatics points). All participants provided written informed consent. The study was approved by the Ethics Committee of South China Normal University (Approval No. SCNU-SPT-2025-025) and was conducted in accordance with the Declaration of Helsinki.

**TABLE 1:** Characteristics of the 24 male competitive swimmers (mean ± SD).

Characteristic	Mean ± SD
Age (years)	21.53 ± 2.43
Stature (m)	1.82 ± 0.06
Body mass (kg)	77.82 ± 6.51
Arm span (m)	1.90 ± 0.07
Training experience (years)	9.52 ± 2.78
Weekly training volume (km)	42.00 ± 8.00
50-m freestyle season best (s)	24.01 ± 0.60
World aquatics points	745.12 ± 35.44

### 2.2 Instrumentation

After a standardised self-selected warm-up, participants performed maximal-effort 50-m front crawl trials without breathing, so that head and trunk configuration remained as consistent as possible. Hand kinetics were recorded bilaterally with eo SwimBETTER handsets (eo Lab, Sydney, Australia). According to the manufacturer, each handset combines a pressure sensor, which measures the pressure differential between the palm and the back of the hand, with a tri-axial accelerometer and gyroscope that track hand orientation and movement. Force is derived from the pressure differential together with the swimmer’s hand length and width entered at set-up, and the system reports the resultant hand force, its forward (propulsive), lateral and vertical components in a pool-referenced frame, and hand velocity (eo Lab, 2025). The two handsets operate as separate units. The specific handset model and firmware version were not disclosed by the manufacturer, which also does not publish the native sampling rate, calibration procedure or force-derivation algorithm; the exported series used here were on the 100-Hz time base of the kinematic frames. The device’s validation study reported no significant between-session differences in its kinetic variables and agreement with an external reference for stroke frequency, but it did not test its force outputs against an independent force criterion (Bastián-Antón et al., 2025). We therefore treat the device output as the operational reference and describe the estimand throughout as reconstruction of SwimBETTER-derived kinetics, not of an independently validated hydrodynamic force. Because no defensible criterion error model is available, reference uncertainty is stated as a limitation rather than propagated numerically.

Three-dimensional kinematics were captured with a Qualisys system (Oqus 700+ underwater and above-water cameras; Qualisys AB, Gothenburg, Sweden) sampling at 100 Hz in a calibrated volume of 10.0 × 3.0 × 2.0 m (15 cameras, calibration residual <0.8 mm; Figure 1). Fourteen retroreflective markers were placed bilaterally over seven landmarks of the upper limb and trunk (Figure 2A; Table 2), all by the same investigator. The origin of the laboratory vertical axis lay at the water surface (Z = 0); fingertip crossings of this level coincided with the onset and offset of each hand’s force record.

**FIGURE 1:**
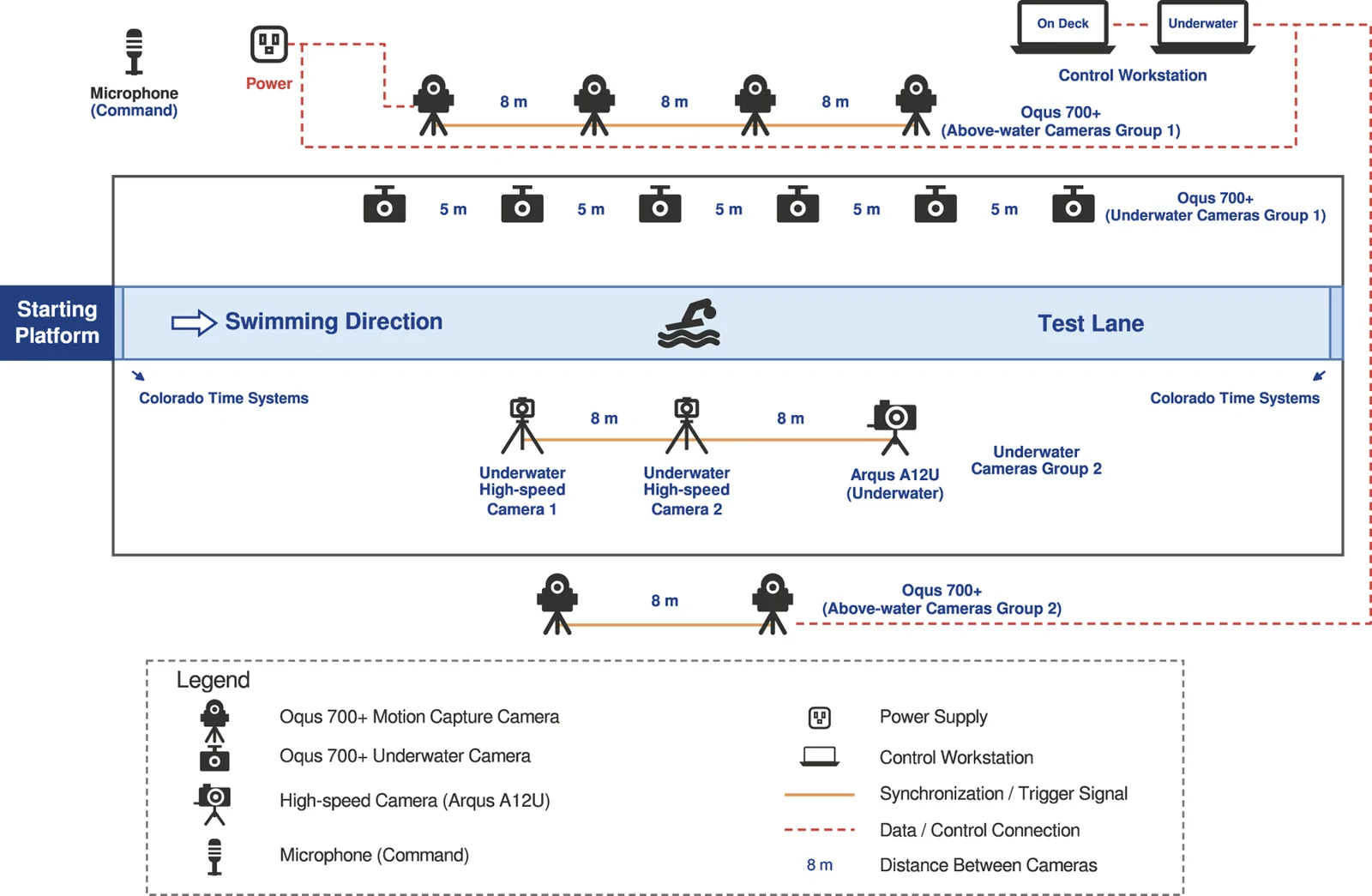
Layout of the underwater and above-water motion-capture system in the test lane.

**FIGURE 2:**
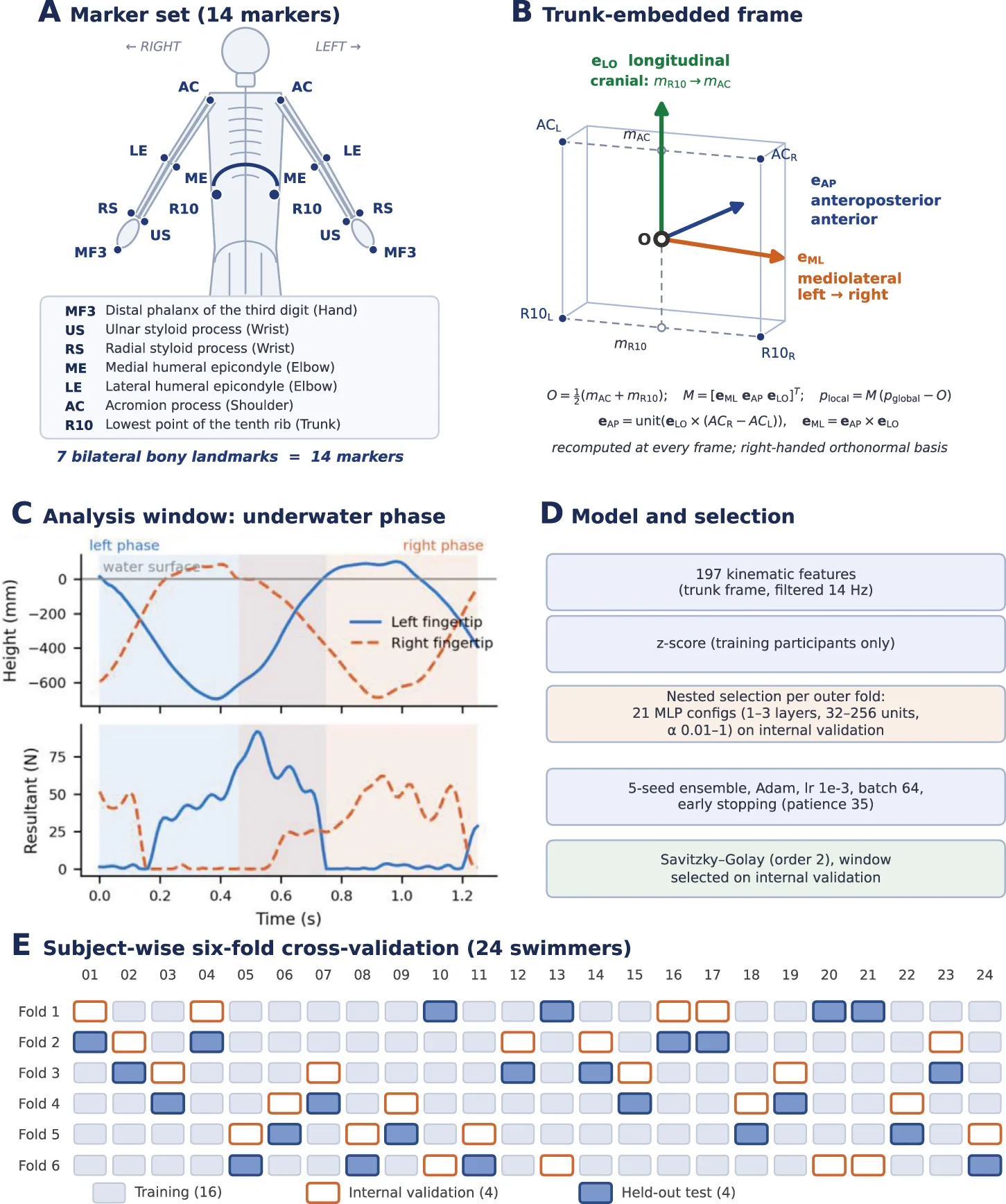
Measurement and modelling pipeline. **(A)** Fourteen retroreflective markers on seven bilateral landmarks. **(B)** Trunk-embedded reference frame, recomputed at every frame. **(C)** Analysis window, shown for one example swimmer: the fingertip (MF3) height of each hand (top) and the resultant force of each hand (bottom). The shaded areas mark the underwater phase of each hand, from fingertip entry (downward crossing of the water surface, Z = 0) to exit; each hand’s force record is aligned with that handset’s own synchronisation offset. **(D)** Model, nested selection and post-processing. **(E)** Subject-wise six-fold cross-validation: in each fold four swimmers were held out for testing (filled), four formed the internal validation set used for architecture, regularisation and smoothing selection and for early stopping (outlined), and sixteen were used for fitting.

**TABLE 2:** Bony landmarks.

Segment	Bony landmark	Abbreviation	Side
Hand	Distal phalanx of the third digit	MF3	Left, right
Wrist	Ulnar styloid process	US	Left, right
Wrist	Radial styloid process	RS	Left, right
Elbow	Medial humeral epicondyle	ME	Left, right
Elbow	Lateral humeral epicondyle	LE	Left, right
Shoulder	Acromion process	AC	Left, right
Trunk	Lowest point of the tenth rib	R10	Left, right

### 2.3 Stroke-phase definition and synchronisation

Following the conventional segmentation of the front crawl arm stroke into an underwater phase (entry and catch, pull, push) and an aerial recovery (Chollet et al., 2000), and the practice of analysing hand kinetics over the underwater phase only (Santos et al., 2022), the analysis window for each hand was its underwater phase: from the frame at which the third-digit (MF3) marker crossed the water surface downwards (entry, event X1) to the frame at which it crossed upwards (exit, event X4). For every swimmer, one complete, fully in-volume underwater phase of each hand from the same trial was analysed, giving 48 hand-specific windows (24 per hand; 60–101 frames, median 78; 3,766 frames in total). All events were identified from kinematics before any force or model output was examined. The capture data were exported as one worksheet per hand-specific underwater phase (48 worksheets; the two windows of a trial overlap by 13–39 frames), and the correspondence between each analysed window, its export worksheet, its event times and its device offset is listed in the Supplementary Data (window map). Two recordings found to be exact duplicates of other trials were excluded before analysis.

Because the two handsets are independent units, the device record of each hand was aligned to the kinematics separately. The X1 and X4 events of that hand were located in both the kinematic and the device record, and the device time series was shifted by the resulting offset (for the 12 swimmers whose event times were logged in both systems, the offsets obtained at X1 and X4 were identical). Each hand’s kinetics were therefore used only in the window aligned with that hand’s own offset. Synchronisation accuracy was assessed in three ways (results in Section 3.7). First, where the two windows of a trial overlapped, we compared the offsets obtained independently for the two handsets. Second, we tested physical consistency: the share of measured impulse recorded while the fingertip was more than 30 mm above the water, and the shift of the force record that minimised this share. Third, we cross-correlated the device-reported hand velocity with fingertip speed computed from the markers. The primary target was re-analysed after re-aligning the force records by the median lag from the third check (sensitivity analysis).

### 2.4 Target variables and signal processing

For each hand the device provided the resultant force and its forward (propulsive), lateral and vertical components, together with hand velocity; the resultant equalled the Euclidean norm of the three components (r = 1.00, mean difference −0.05 N). Six targets were modelled per hand: resultant, propulsive, lateral and vertical force, and two operational, device-derived power indices. The total power index was the product of the resultant force magnitude and the device-reported hand speed, and the propulsive power index the product of the forward force component and the same hand speed. Because neither is the scalar (dot) product of the force and hand-velocity vectors, they are not mechanical power in the physical sense and are not interpreted as the rate of work done on the water or against drag; they are reported only as operational quantities. Because both indices are computed deterministically from force and hand speed, and hand speed is closely related to kinematic predictors, they are not independent evidence of generalisation beyond the force channels. Resultant force was the primary target and carried the full agreement analysis; the other five targets were evaluated at the waveform and descriptor levels.

Of the 52,724 marker-frames, 474 (0.90%) had a missing coordinate triplet. All gaps occurred in the contralateral (recovering) arm near the start or end of a window, in 10 of the 48 windows (20 gaps; median 26, maximum 47 frames), and none affected the markers of the analysed hand. Gaps were filled by linear interpolation within the continuous trial record, and a sensitivity analysis excluded every window containing any interpolated frame. Trajectories were then low-pass filtered with a zero-lag fourth-order Butterworth filter at 14 Hz. The cut-off was chosen by residual analysis (Winter 2009): the median of the marker-specific and axis-specific optimal cut-offs was 14 Hz (IQR 14–15 Hz). Filtering, numerical differentiation and feature lags were applied to the continuous trial record (1.09–1.72 s) before the windows were extracted, so window edges did not require padding; only lags at the ends of the trial record were edge-replicated. For statistics, each window was time-normalised to 101 points (0%–100% of the underwater phase); models were fitted on the original 100-Hz frames.

### 2.5 Feature construction

A right-handed trunk-embedded frame was reconstructed at every frame from the two acromion and two tenth-rib markers (Figure 2B; Table 3). The frame removes the swimmer’s position and orientation in the capture volume but is not scaled to body size, so anthropometric differences remain in the inputs. Within this frame, 197 features in nine families were derived (Table 4). Velocities and accelerations were obtained by central differences of the filtered laboratory trajectories and projected onto the trunk axes; hand velocity relative to the trunk was obtained by differentiating the local coordinates. The hand-plane normal is the unit cross-product of the ulnar-to-radial styloid vector and the wrist-to-fingertip vector. The angle between this normal and the hand velocity vector is reported as a geometric hand-plane–velocity angle rather than a hydrodynamic angle of attack, which would require the local flow velocity.

**TABLE 3:** Trunk-embedded coordinate system, reconstructed at every frame.

Element	Definition	Positive direction
Shoulder mid-point	m_AC_ = (AC_L_ + AC_R_)/2	—
Rib mid-point	m_R10_ = (R10_L_ + R10_R_)/2	—
Frame origin	O = (m_AC_ + m_R10_)/2	—
Longitudinal axis	e_LO_ = (m_AC_ − m_R10_)/‖m_AC_ − m_R10_‖	Cranial
Anteroposterior axis	e_AP_ = e_LO_ × (AC_R_ − AC_L_)/‖e_LO_ × (AC_R_ − AC_L_)‖	Anterior
Mediolateral axis	e_ML_ = e_AP_ × e_LO_	Left to right
Local coordinates	p_local_ = R^T^(p_global_ − O), R = [e_ML_ e_AP_ e_LO_]	—

‖·‖, Euclidean norm; ×, vector cross-product. The frame is not scaled to body size. For right-hand samples all mediolateral components were sign-inverted and ipsilateral/contralateral roles exchanged.

**TABLE 4:** Composition of the 197-dimensional feature set and model-specific sensitivity of validation R^2^ to grouped permutation of each family (resultant force; internal validation sets of the six outer folds).

Feature family	n	Composition	Loss of validation R^2^ when the family is permuted, mean (range across folds)
Segment orientations	18	Unit direction vectors of upper arm, forearm and hand (ipsilateral and contralateral) in the trunk frame	0.019 (−0.010–0.081)
Linear accelerations	12	Ipsilateral hand, wrist and elbow acceleration (magnitude and three components)	0.026 (−0.012–0.050)
Segment lengths/anthropometry	10	Biacromial width, trunk length, and upper-arm, forearm and hand length and wrist breadth of both arms (trial medians)	0.002 (−0.029–0.037)
Linear velocities	62	Ipsilateral hand, wrist and elbow velocity, hand velocity relative to the trunk, contralateral hand velocity, plus ±2 and ±4 frame lags of the principal hand variables	0.532 (0.367–0.660)
Hand-plane normal	6	Unit normal of the plane spanned by the ulnar→radial styloid and wrist→fingertip vectors (both hands)	0.014 (−0.008–0.039)
Angular velocities	3	Forearm angular speed (both arms) and trunk angular speed	−0.002 (−0.006 to 0.003)
Hand-plane–velocity angle	2	Geometric angle between the hand-plane normal and the hand velocity vector (both hands)	−0.003 (−0.020 to 0.004)
Joint angles	18	Elbow, wrist and shoulder-elevation angles of both arms, plus ±2 and ±4 frame lags of the ipsilateral angles	−0.006 (−0.037 to 0.018)
Marker positions in trunk frame	66	Coordinates of all 14 markers, plus ±2 frame lags of four ipsilateral hand/wrist/elbow markers	0.096 (−0.038–0.195)

All features of a family were permuted jointly (five repeats per fold). Values describe the model-specific sensitivity of validation R^2^ to grouped permutation; they preserve relationships within a family but are not conditional importances and do not isolate information unique to a family given correlated families. Negative values indicate that validation performance improved after permutation, not that the family carries no unique information.

### 2.6 Model, nested selection and benchmarks

Right-hand samples were mirrored by inverting all mediolateral components and exchanging the ipsilateral and contralateral roles, so that a single model served both hands (3,766 single-hand samples; Figure 2C). The same model family was trained independently for each of the six targets. Networks were fully connected feed-forward regressors with rectified linear units, trained in scikit-learn 1.8.0 (Pedregosa et al., 2011) with Adam (Kingma and Ba, 2015; learning rate 1 × 10^−3^, batch size 64) and L2 regularisation. Inputs and outputs were z-standardised with training-participant statistics only. Training stopped when the internal-validation mean squared error had not improved for 35 epochs, and the best-epoch weights were restored. Predictions were averaged over a five-network ensemble (seeds 0–4; Lakshminarayanan et al., 2017) and smoothed with a second-order Savitzky–Golay filter (Savitzky and Golay, 1964; Figure 2D).

Model selection was nested (Varma and Simon, 2006). Participants were randomly allocated (fixed seed) to six groups of four. In each outer fold one group was held out for testing, the next group formed the internal validation set, and the remaining sixteen swimmers were used for fitting (Figure 2E). Inside each fold, 21 configurations were compared on the internal validation set: one to three hidden layers (64, 128, 256, 64–32, 128–64, 256–128 or 128–64–32 units) crossed with α ∈ {0.01, 0.1, 1.0}. The best configuration, selected on the primary target, was used for all six targets in that fold. The Savitzky–Golay window (none, 11, 15, 21, 25 or 31 frames) was then selected on the same internal validation set. No frame from a test participant contributed to fitting or to any selection, and the configuration was locked before the fold’s test swimmers were predicted (per-fold choices in Section 3.6; logs in Supplementary Data).

Six comparators (four alternative learned models and two baselines) were evaluated for the primary target under identical folds, standardisation and post-processing: (1) a group-mean null model; (2) a phase-conditioned template that predicts, for each held-out window, the mean training waveform at the same normalised phase and therefore carries no swimmer-specific information; (3) ridge regression on the marker coordinates; (4) ridge regression on the full feature set, with penalties chosen by generalised cross-validation within each training set; (5) histogram-based gradient boosting on the full feature set, with a search budget matched to the network (21 configurations of learning rate, maximum leaf nodes and minimum leaf size, and the number of boosting iterations chosen on the internal validation set); and (6) the same network restricted to marker coordinates, with its own 21-configuration nested search.

### 2.7 Statistical analysis

#### 2.7.1 Unit of analysis

Each swimmer contributed one underwater phase per hand, so every hand-specific analysis has n = 24 independent participants. Where statistics pool time-normalised samples (24 × 101 per hand; ICC, pooled correlations, regression slopes and Bland–Altman analysis), confidence intervals were obtained with a participant-cluster bootstrap (2,000 resamples), which resamples swimmers rather than time points. Analyses used Python 3.11 with SciPy 1.17.1, spm1d 0.4.54, statsmodels and pingouin 0.6.1.

#### 2.7.2 Tier 1 all targets

For each window we computed the RMSE, the RMSE normalised to the measured range of that window (nRMSE) and the Pearson correlation; correlations were pooled by Fisher z with t-based 95% CIs across participants. Waveform similarity was quantified with the coefficient of multiple correlation for synchronously measured waveforms (CMC; Kadaba et al., 1989; Ferrari et al., 2010) and with ICC(A,1) (McGraw and Wong, 1996; Koo and Li, 2016). Waveform-level inference used one-dimensional statistical parametric mapping (SPM) paired t-tests (Pataky, 2012; Pataky et al., 2015), with random-field-theory and 5,000-permutation inference (α = 0.05, two-tailed). Residual normality was rejected for all targets, so the non-parametric result is primary. The mean, peak and minimum of each window were compared with paired t-tests, with Holm correction (Holm, 1979) across the 36 tests.

#### 2.7.3 Tier 2 primary target

For resultant force we additionally report RMSE with a participant-bootstrap CI; pooled Pearson and Spearman correlations; the repeated-measures correlation, which isolates within-swimmer association (Bakdash and Marusich, 2017); ordinary-least-squares and Deming regression with an error-variance ratio λ = 1, because no independent estimate of the ratio of the two methods’ error variances was available; and Bland–Altman analysis for multiple observations per individual with a varying true value (Bland and Altman, 1986; 2007). In that analysis the limits of agreement use the total SD from a one-way random-effects decomposition of the differences, and proportional bias is the slope of the difference on the mean. Four discrete variables were extracted from each window: peak force, impulse (trapezoidal integral), mean force and time to peak (% of the underwater phase). They were compared with paired t-tests, or Wilcoxon signed-rank tests when Shapiro–Wilk p < 0.05, with Cohen’s dz and the between-participant correlation. For time to peak, both the signed bias and the distribution of absolute errors are reported.

#### 2.7.4 Equivalence and multiplicity

The ±10% equivalence margins are a practical convention rather than a margin derived from a device error budget, which is unavailable, and should be read as such. For time to peak the margin was ±10 percentage points of the underwater phase. Because the analysis plan was not preregistered, no test is labelled confirmatory. Two one-sided tests (TOST; Lakens, 2017) are reported with Holm correction across the eight discrete-variable tests, and all other families are described as exploratory. A non-significant difference test is reported as a failure to detect a difference, never as evidence of equivalence.

#### 2.7.5 Inter-limb symmetry

For mean force, impulse and peak force, the symmetry index SI = 200 × (L − R)/(L + R) was computed separately from measured and estimated values. Agreement was quantified with the bias and limits of agreement, the mean absolute error, Pearson r, ICC(A,1) and the percentage of swimmers for whom the dominant side was identified correctly.

#### 2.7.6 Variance decomposition and model interpretation

The variance of the measured waveforms was split into within-window and between-participant components, and agreement was evaluated separately for each. Model-specific sensitivity to grouped permutation was computed on each fold’s internal validation set as the loss of R^2^ when all features of a family were permuted jointly (five repeats). This preserves relationships within a family, but it is not a conditional importance measure (Strobl et al., 2008) and does not isolate the information unique to a family given correlated families. Fold-to-fold ranges are reported. Participant-level hand length, wrist breadth and their product were correlated with measured mean force and, after adjustment for the mean of the two methods, with the residual amplitude error.

#### 2.7.7 Sensitivity analyses

Besides the synchronisation and interpolation checks, the complete pipeline was run under a broader window definition in which both hands were modelled in every window, so that each hand’s samples also covered part of its recovery and entry.

#### 2.7.8 Reproducibility

All steps from the raw export to the tables and figures are implemented in a single set of scripts. The scripts, the out-of-fold predictions for every frame and target, the fold assignments and the per-fold selection logs are provided as Supplementary Data. No single deployable model trained on all participants is released, because device-off use has not been validated (Section 4).

## 3 Results

### 3.1 Benchmark comparison

All learned models and the template outperformed the group-mean null model (Table 5). The null model’s R^2^ is slightly negative because its training-set mean differs from the mean of each held-out fold. The proposed network (resultant force: RMSE 24.5 ± 6.1 N left, 23.3 ± 9.9 N right) was more accurate than ridge regression on the full feature set (paired ΔRMSE −7.0 N on both sides, p ≤ 0.001), which suffered from its many correlated inputs. It was statistically indistinguishable from gradient boosting given the same search budget (ΔRMSE −0.4 and +1.3 N; 95% CIs spanning zero), from ridge regression on marker coordinates (+0.3 and −0.3 N) and from the same network restricted to coordinates (+0.4 and −0.7 N). The phase-conditioned template, which knows only the typical shape of the underwater force curve, was the most accurate model: RMSE 21.8 ± 6.4 and 21.2 ± 7.6 N, per-window r 0.894 and 0.869, CMC 0.869 and 0.837. The network’s RMSE was 2.7 N (95% CI 0.9–4.5; p = 0.010) higher than the template’s for the left hand and 2.1 N (−0.2 to 4.5; p = 0.121) higher for the right hand. Thus neither the engineered feature set nor the network architecture was necessary to reach the accuracy of the learned models, and none of the learned models improved waveform accuracy beyond a template.

**TABLE 5:** Benchmark comparison for resultant force under identical subject-wise six-fold cross-validation, nested selection and post-processing.

Model	Hand	R^2^ (fold mean ± SD, n = 6)	RMSE, N (mean ± SD, n = 24)	Per-window r	CMC	ΔRMSE, proposed − model, N [95% CI]	p
Group mean (null)	Left	−0.030 ± 0.029	36.7 ± 8.5	—	0.070	−12.2 [−14.4, −9.9]	<0.001
Group mean (null)	Right	−0.068 ± 0.074	30.8 ± 7.8	—	0.065	−7.5 [−11.4, −3.1]	0.005
Phase-conditioned template	Left	0.625 ± 0.077	21.8 ± 6.4	0.894	0.869	2.7 [0.9, 4.5]	0.010
Phase-conditioned template	Right	0.420 ± 0.246	21.2 ± 7.6	0.869	0.837	2.1 [−0.2, 4.5]	0.121
Ridge, marker coordinates	Left	0.546 ± 0.085	24.2 ± 6.5	0.843	0.831	0.3 [−1.8, 2.5]	0.900
Ridge, marker coordinates	Right	0.295 ± 0.350	23.7 ± 8.7	0.839	0.789	−0.3 [−3.5, 2.4]	0.527
Ridge, full set	Left	0.172 ± 0.435	31.5 ± 13.0	0.801	0.771	−7.0 [−12.0, −3.0]	0.001
Ridge, full set	Right	−0.095 ± 0.362	30.3 ± 9.9	0.769	0.712	−7.0 [−11.5, −3.2]	<0.001
Gradient boosting, full set	Left	0.520 ± 0.071	24.8 ± 6.7	0.848	0.807	−0.4 [−1.6, 1.0]	0.565
Gradient boosting, full set	Right	0.381 ± 0.231	22.0 ± 8.3	0.853	0.790	1.3 [−0.8, 3.5]	0.241
ANN, marker coordinates	Left	0.550 ± 0.096	24.1 ± 5.6	0.857	0.830	0.4 [−0.8, 1.6]	0.546
ANN, marker coordinates	Right	0.230 ± 0.455	24.0 ± 10.2	0.839	0.781	−0.7 [−2.4, 1.1]	0.422
**ANN, full set (proposed)**	Left	0.536 ± 0.076	24.5 ± 6.1	0.849	0.830	—	—
**ANN, full set (propo** **sed)**	Right	0.260 ± 0.495	23.3 ± 9.9	0.844	0.793	—	—

Gradient boosting and the coordinates-only network each had a 21-configuration nested search, matching the proposed network. ΔRMSE: paired participant-level difference (negative = proposed network more accurate); 95% CI by participant bootstrap (2,000 resamples); p, Wilcoxon signed-rank. The phase-conditioned baseline predicts, for each held-out window, the mean training waveform at the same normalised phase (0%–100% of the underwater phase) and uses no swimmer-specific information.

Bold, the proposed network (ANN, full feature set).

### 3.2 Waveform accuracy across the six targets

Waveform reconstruction was similar across targets (Table 6; Figures 3, 4). Per-window correlations were 0.84–0.90 for resultant, propulsive and vertical force and for both power indices, and 0.69–0.70 for lateral force. CMC ranged from 0.75 to 0.85 and ICC(A,1) from 0.42 to 0.72, with right-hand agreement generally lower and more variable than left-hand agreement (nRMSE 23.6%–33.8% vs. 21.3%–26.6%). The power indices behaved like their force counterparts, as expected from their construction (Section 2.4).

**TABLE 6:** Waveform-level accuracy for the six kinetic targets (participant level: one underwater phase per hand per swimmer, n = 24 per hand). Force in N, power in W.

Target	Hand	RMSE	nRMSE (%)	Per-window r (95% CI)	CMC	ICC(A,1) [95% CI]	R^2^
Resultant force	Left	24.5 ± 6.1	21.3 ± 4.5	0.849 (0.793–0.890)	0.830	0.704 [0.67, 0.74]	0.540
Resultant force	Right	23.3 ± 9.9	29.8 ± 21.5	0.844 (0.791–0.884)	0.793^a^	0.639 [0.53, 0.74]	0.345
Propulsive force	Left	22.9 ± 8.6	21.6 ± 6.4	0.899 (0.856–0.930)	0.848	0.724 [0.67, 0.78]	0.579
Propulsive force	Right	22.9 ± 12.1	31.1 ± 19.3	0.873 (0.757–0.936)	0.798^a^	0.609 [0.42, 0.75]	0.292
Lateral force	Left	11.8 ± 4.6	26.6 ± 13.5	0.704 (0.523–0.824)	0.752^a^	0.420 [0.24, 0.58]	0.217
Lateral force	Right	10.0 ± 5.8	23.6 ± 7.3	0.690 (0.525–0.804)	0.766^a^	0.418 [0.22, 0.63]	0.144
Vertical force	Left	18.8 ± 6.5	24.0 ± 11.6	0.823 (0.757–0.873)	0.764	0.640 [0.55, 0.71]	0.457
Vertical force	Right	14.8 ± 5.7	24.2 ± 13.7	0.855 (0.787–0.902)	0.817	0.713 [0.64, 0.78]	0.492
Total power index	Left	102.4 ± 33.3	21.7 ± 5.3	0.864 (0.814–0.902)	0.839	0.720 [0.68, 0.76]	0.575
Total power index	Right	90.2 ± 37.8	29.5 ± 20.1	0.883 (0.837–0.917)	0.802	0.696 [0.60, 0.78]	0.432
Propulsive power index	Left	98.9 ± 37.9	23.7 ± 7.5	0.876 (0.827–0.912)	0.823	0.686 [0.62, 0.75]	0.528
Propulsive power index	Right	95.8 ± 50.8	33.8 ± 19.2	0.853 (0.726–0.924)	0.768^a^	0.560 [0.36, 0.72]	0.191

Per-window correlations pooled by Fisher z (t-based CI, across the 24 participants). CMC, averaged over windows;

^a^ CMC, undefined (negative radicand) for 1–5 windows, which were omitted from the mean. ICC(A,1) on time-normalised samples with a 95% CI, from a participant-cluster bootstrap (2,000 resamples). R2, pooled out-of-fold.

**FIGURE 3:**
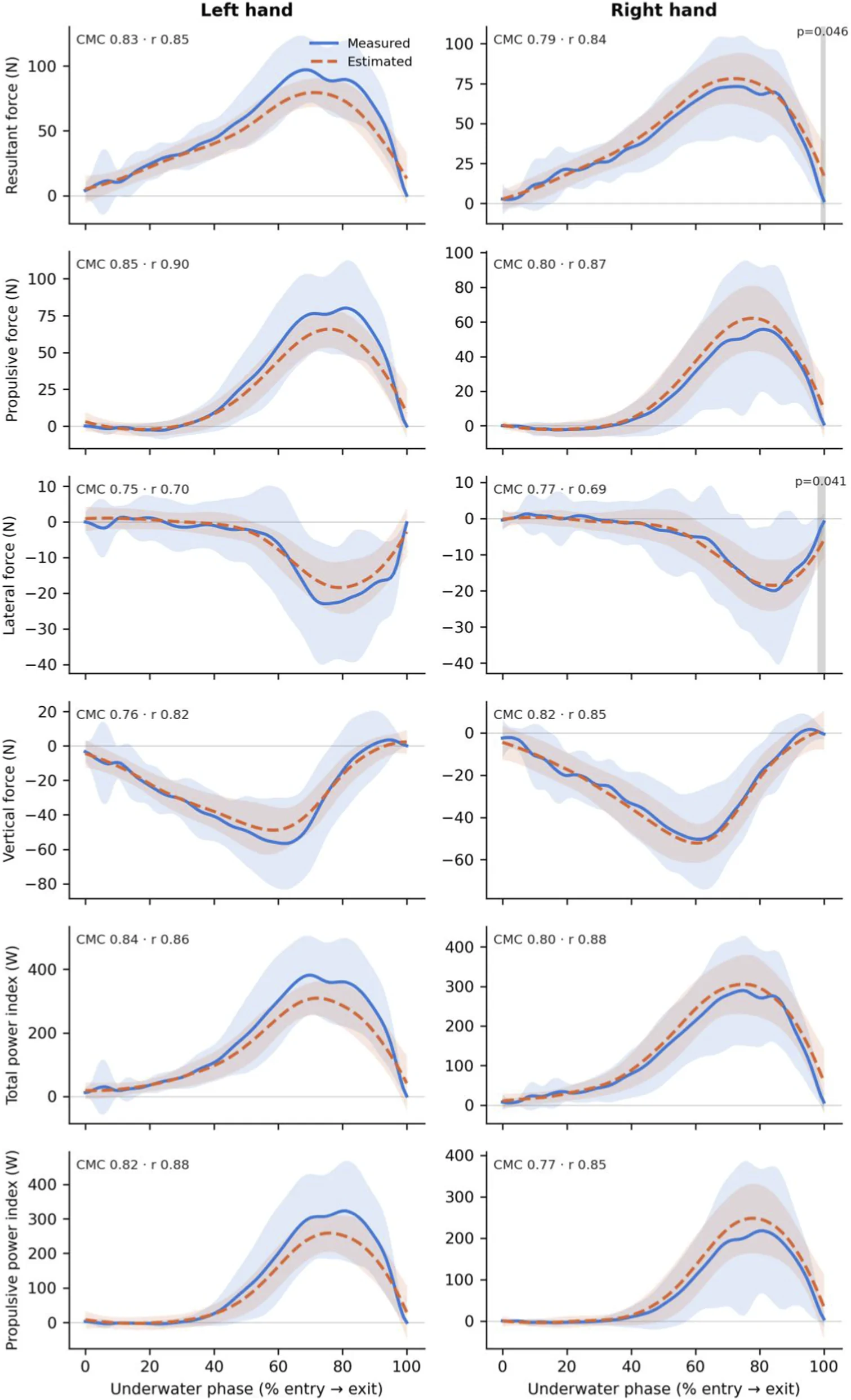
Measured (solid blue) and estimated (dashed orange) waveforms of the six kinetic targets for the left and right hands over the underwater phase (0% = entry, 100% = exit). Lines show the mean and shaded bands ±1 SD across the 24 swimmers. Grey vertical bands mark supra-threshold clusters of the SPM paired t-test (random-field theory, α = 0.05, two-tailed), labelled with the cluster p-value; the non-parametric results are given in Table 7. Values in each panel are the mean CMC and the Fisher-pooled per-window r.

**FIGURE 4:**
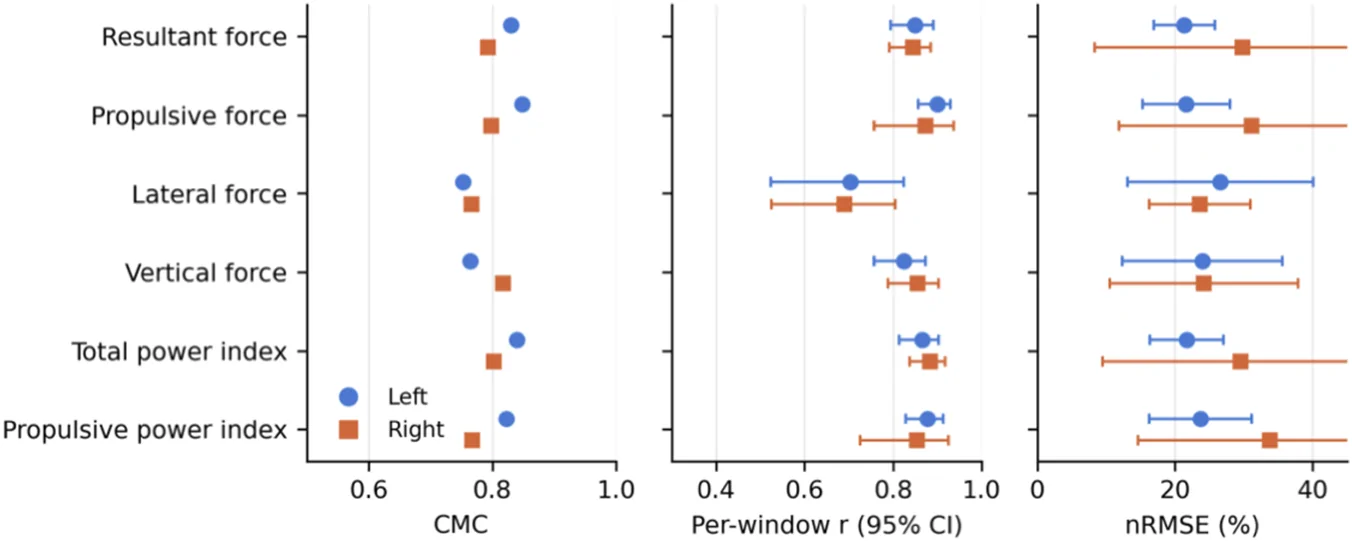
Waveform accuracy for the six targets (n = 24 per hand). Left: mean CMC. Centre: Fisher-pooled per-window correlation with 95% CI. Right: nRMSE, mean ± SD. Circles, left hand; squares, right hand.

Non-parametric SPM detected no supra-threshold cluster for ten of the twelve target–hand combinations (Table 7). The exceptions were right-hand resultant and lateral force, each with a narrow cluster at 98%–100% of the underwater phase (p = 0.025 and 0.004), where the estimate did not fall to zero as sharply as the measured force at exit (Figure 3). These are failures to detect a difference with 24 swimmers per hand, not evidence of equivalence.

**TABLE 7:** Waveform-level inference: SPM paired t-tests, estimated versus measured (n = 24 per hand, 101 nodes).

Target	Hand	Residual normality	Critical t (t*)	Maximum absolute t	Parametric (RFT) supra-threshold cluster, % of phase (p)	Non-parametric (permutation) supra-threshold cluster, % of phase (p)
Resultant force	Left	Violated	3.51	3.18	None	None
Resultant force	Right	Violated	3.44	3.77	99%–100% (p = 0.046)	98%–100% (p = 0.025)
Propulsive force	Left	Violated	3.34	2.74	None	None
Propulsive force	Right	Violated	3.42	2.49	None	None
Lateral force	Left	Violated	3.46	3.05	None	None
Lateral force	Right	Violated	3.53	5.80	98%–100% (p = 0.041)	98%–100% (p = 0.004)
Vertical force	Left	Violated	3.49	1.84	None	None
Vertical force	Right	Violated	3.46	2.03	None	None
Total power index	Left	Violated	3.54	2.83	None	None
Total power index	Right	Violated	3.45	3.25	None	None
Propulsive power index	Left	Violated	3.33	2.41	None	None
Propulsive power index	Right	Violated	3.39	2.03	None	None

Random-field-theory (parametric) and 5,000-permutation (non-parametric) inference, α = 0.05 two-tailed. Because residual normality was rejected for every target, the non-parametric result is treated as primary. “None” indicates that no difference was detected at this sample size; it does not establish equivalence.

At the descriptor level (Table 8), estimated mean values were not detectably different from measured ones after Holm correction. Extremes were compressed. Left-hand peak resultant force was underestimated by 32.6 N (Holm p < 0.001), as were the left-hand peaks of propulsive force and of both power indices, the lateral-force peaks of both hands and the left-hand lateral and vertical minima (Holm p ≤ 0.039). The other right-hand extremes were not detectably different. The between-swimmer SD of every estimated descriptor was markedly smaller than the measured SD, so estimates were shrunk towards the group mean.

**TABLE 8:** Window-level mean, peak and minimum of each kinetic target, measured versus estimated (n = 24 per hand; each descriptor extracted from the 100-Hz record of each window).

Target	Hand	Mean, meas.	Mean, est.	p (Holm)	Peak, meas.	Peak, est.	p (Holm)	Min, meas.	Min, est.	p (Holm)
Resultant force	Left	51.8 ± 10.6	44.1 ± 6.4	0.002 (0.061)	116.0 ± 20.9	83.4 ± 10.5	<0.001 (<0.001)	0.3 ± 0.8	−0.5 ± 9.4	0.673 (1.000)
Resultant force	Right	40.3 ± 12.1	43.4 ± 8.0	0.351 (1.000)	90.2 ± 29.9	82.7 ± 16.8	0.320 (1.000)	0.0 ± 0.0	−1.1 ± 3.8	0.172 (1.000)
Propulsive force	Left	30.7 ± 11.1	25.1 ± 5.6	0.043 (1.000)	99.8 ± 26.8	68.2 ± 12.9	<0.001 (0.004)	−6.3 ± 6.4	−5.5 ± 6.7	0.692 (1.000)
Propulsive force	Right	19.2 ± 13.9	22.5 ± 7.6	0.285 (1.000)	70.9 ± 31.7	65.5 ± 18.7	0.512 (1.000)	−10.6 ± 20.0	−5.5 ± 4.7	0.238 (1.000)
Lateral force	Left	−7.4 ± 5.5	−5.9 ± 2.4	0.282 (1.000)	10.5 ± 8.6	4.0 ± 3.6	0.001 (0.039)	−36.2 ± 10.9	−20.6 ± 5.8	<0.001 (<0.001)
Lateral force	Right	−5.6 ± 3.7	−6.1 ± 2.6	0.542 (1.000)	12.7 ± 12.9	2.6 ± 2.1	<0.001 (0.027)	−29.9 ± 13.0	−20.3 ± 6.1	0.004 (0.100)
Vertical force	Left	−27.0 ± 9.8	−25.3 ± 5.7	0.472 (1.000)	9.8 ± 15.9	4.7 ± 6.8	0.175 (1.000)	−73.6 ± 23.4	−53.4 ± 10.6	0.001 (0.039)
Vertical force	Right	−24.2 ± 6.9	−25.7 ± 4.6	0.361 (1.000)	4.9 ± 7.0	4.3 ± 6.4	0.722 (1.000)	−62.7 ± 20.5	−55.7 ± 9.2	0.102 (1.000)
Total power index	Left	174.9 ± 50.5	144.7 ± 27.8	0.005 (0.129)	479.2 ± 115.6	326.3 ± 59.1	<0.001 (<0.001)	0.8 ± 2.5	−12.4 ± 35.0	0.076 (1.000)
Total power index	Right	130.7 ± 46.7	143.9 ± 30.1	0.266 (1.000)	357.8 ± 128.5	326.1 ± 75.8	0.338 (1.000)	0.0 ± 0.0	−8.0 ± 22.0	0.087 (1.000)
Propulsive power index	Left	119.4 ± 45.0	97.0 ± 26.2	0.050 (1.000)	418.9 ± 132.6	273.9 ± 63.4	<0.001 (0.006)	−11.7 ± 15.8	−24.3 ± 28.1	0.111 (1.000)
Propulsive power index	Right	72.6 ± 53.7	87.6 ± 30.1	0.240 (1.000)	285.6 ± 134.6	265.1 ± 77.4	0.568 (1.000)	−31.6 ± 94.0	−20.6 ± 22.3	0.582 (1.000)

Paired t-tests; Holm adjustment across the 36 tests of this table (exploratory family).

### 3.3 Primary target: error and agreement

For resultant force, RMSE was 24.5 N (95% CI 22.1–27.0) for the left hand and 23.3 N (19.8–27.2) for the right, and ranged across swimmers from 14.3 to 36.1 N and from 11.0 to 49.4 N (Table 9). Pooled over time-normalised samples, the correlation was 0.763 (0.713–0.817) and 0.646 (0.547–0.756). The repeated-measures correlation, which reflects within-swimmer association only, was 0.799 and 0.754. ICC(A,1) was 0.704 (0.665–0.743) and 0.639 (0.528–0.738). Regression slopes were below unity (OLS 0.550 and 0.577; Deming 0.654 (0.590–0.743) and 0.841 (0.682–1.120)), so the estimated range was compressed, most clearly for the left hand (Figure 5).

**TABLE 9:** Primary target (resultant force): error, association and agreement.

Statistic	Left hand	Right hand
RMSE, N: mean ± SD [95% CI]	24.5 ± 6.1 [22.1, 27.0]	23.3 ± 9.9 [19.8, 27.2]
RMSE range, N	14.3–36.1	11.0–49.4
nRMSE, %	21.3 ± 4.5	29.8 ± 21.5
Pooled Pearson r [cluster-bootstrap 95% CI]	0.763 [0.713, 0.817]	0.646 [0.547, 0.756]
Pooled Spearman ρ	0.793	0.701
Repeated-measures correlation r [95% CI]	0.799 [0.78, 0.81]	0.754 [0.74, 0.77]
ICC(A,1) [cluster-bootstrap 95% CI]	0.704 [0.665, 0.743]	0.639 [0.528, 0.738]
OLS slope [95% CI]	0.550 [0.505, 0.605]	0.577 [0.494, 0.691]
Deming slope, λ = 1 [95% CI]	0.654 [0.590, 0.743]	0.841 [0.682, 1.120]

RMSE, statistics are over the 24 windows per hand. Pooled statistics use 24 × 101 time-normalised samples per hand; their confidence intervals come from a participant-cluster bootstrap (2,000 resamples), which respects the dependence among time points of the same swimmer. The repeated-measures correlation (Bakdash and Marusich, 2017) quantifies within-swimmer association after removing between-swimmer offsets.

**FIGURE 5:**
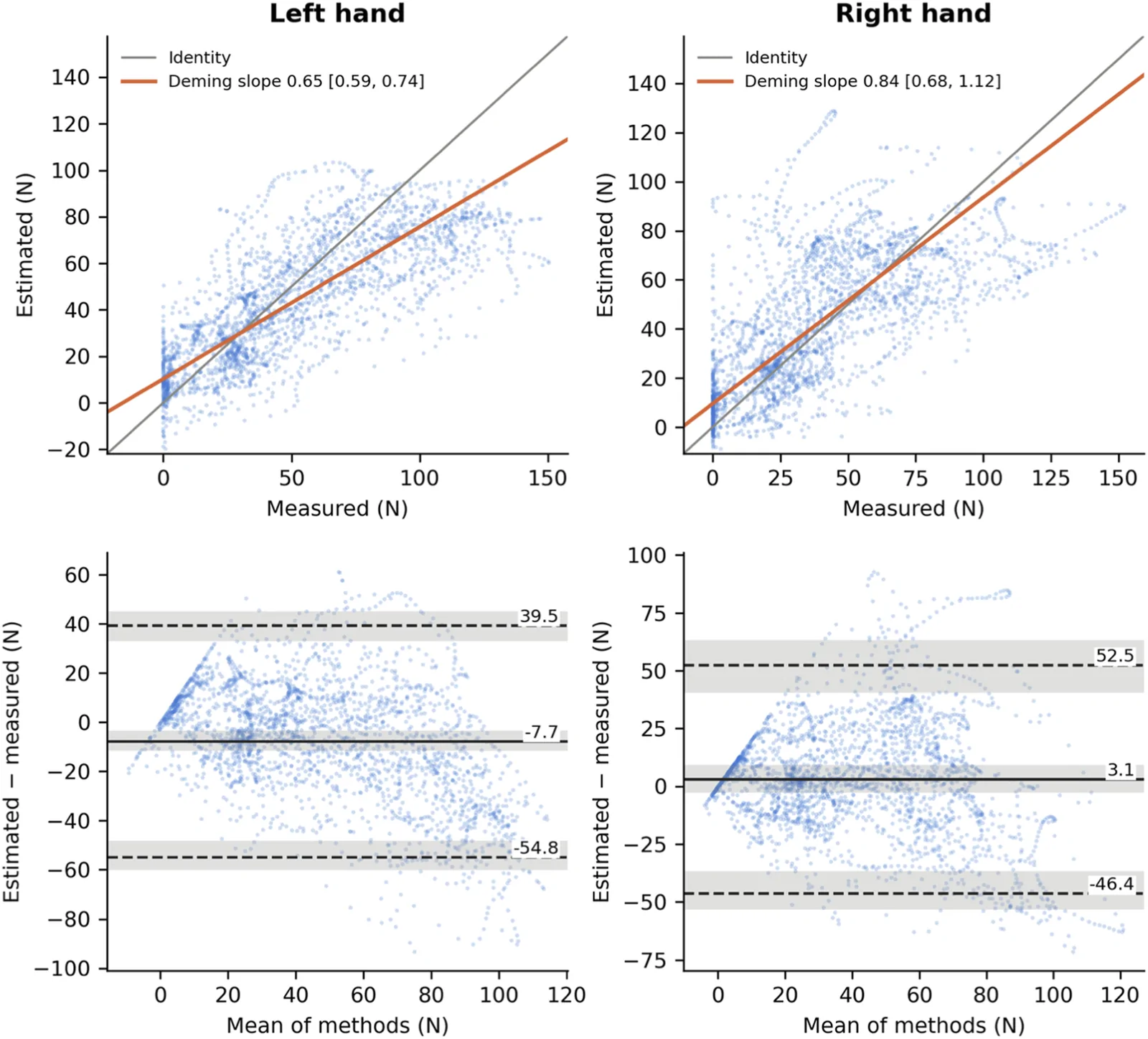
Primary target (resultant force). Top: estimated versus measured force for all time-normalised samples (24 swimmers × 101 points per hand), with the identity line (grey) and the Deming regression line (orange, λ = 1; slope and participant-cluster bootstrap 95% CI in the legend). Bottom: Bland–Altman plots. The solid line is the bias and the dashed lines are the 95% limits of agreement from the variance-components method; grey bands show their participant-cluster bootstrap 95% CIs. Points are not independent within a swimmer; all intervals account for this clustering.

Bland–Altman analysis (Table 10; Figure 5) showed a bias of −7.7 N (−11.7 to −3.3) for the left hand and +3.1 N (−2.7–9.2) for the right. The limits of agreement were −54.8 to 39.5 N and −46.4 to 52.5 N, against measured peaks of about 90–116 N. Of the variance of the differences, a between-swimmer component (SD 10.6 and 15.8 N) accompanied a larger within-window component (SD 21.6 and 19.7 N). Proportional bias was significant for the left hand (slope −0.37 (−0.46 to −0.26)) but not for the right (−0.14 [−0.31 to 0.09]). The mean paired difference stayed within the ±10%-of-peak band during entry and early pull (about the first half of the underwater phase) for both hands. It then fell below the band for the left hand during the pull–push transition, whereas for the right hand the mean stayed within the band but its 95% confidence interval did not (Figure 6).

**TABLE 10:** Bland–Altman analysis for resultant force with participant-level variance components.

Signal	Bias, N [95% CI]	95% LoA, N	CI of lower LoA, N	CI of upper LoA, N	SD between/within swimmers, N	Proportional-bias slope [95% CI]
Left hand	−7.7 [−11.7, −3.3]	−54.8 to 39.5	−60.0 to −48.2	33.0 to 45.2	10.6/21.6	−0.368 [−0.458, −0.261]
Right hand	3.1 [−2.7, 9.2]	−46.4 to 52.5	−53.4 to −36.8	40.4 to 63.2	15.8/19.7	−0.137 [−0.306, 0.087]

Limits of agreement (LoA) use the total SD, from a one-way random-effects decomposition of the time-normalised differences (Bland and Altman, 2007: multiple observations per individual, true value varying); CIs, from a participant-cluster bootstrap. Negative proportional-bias slopes indicate greater underestimation at higher forces.

**FIGURE 6:**
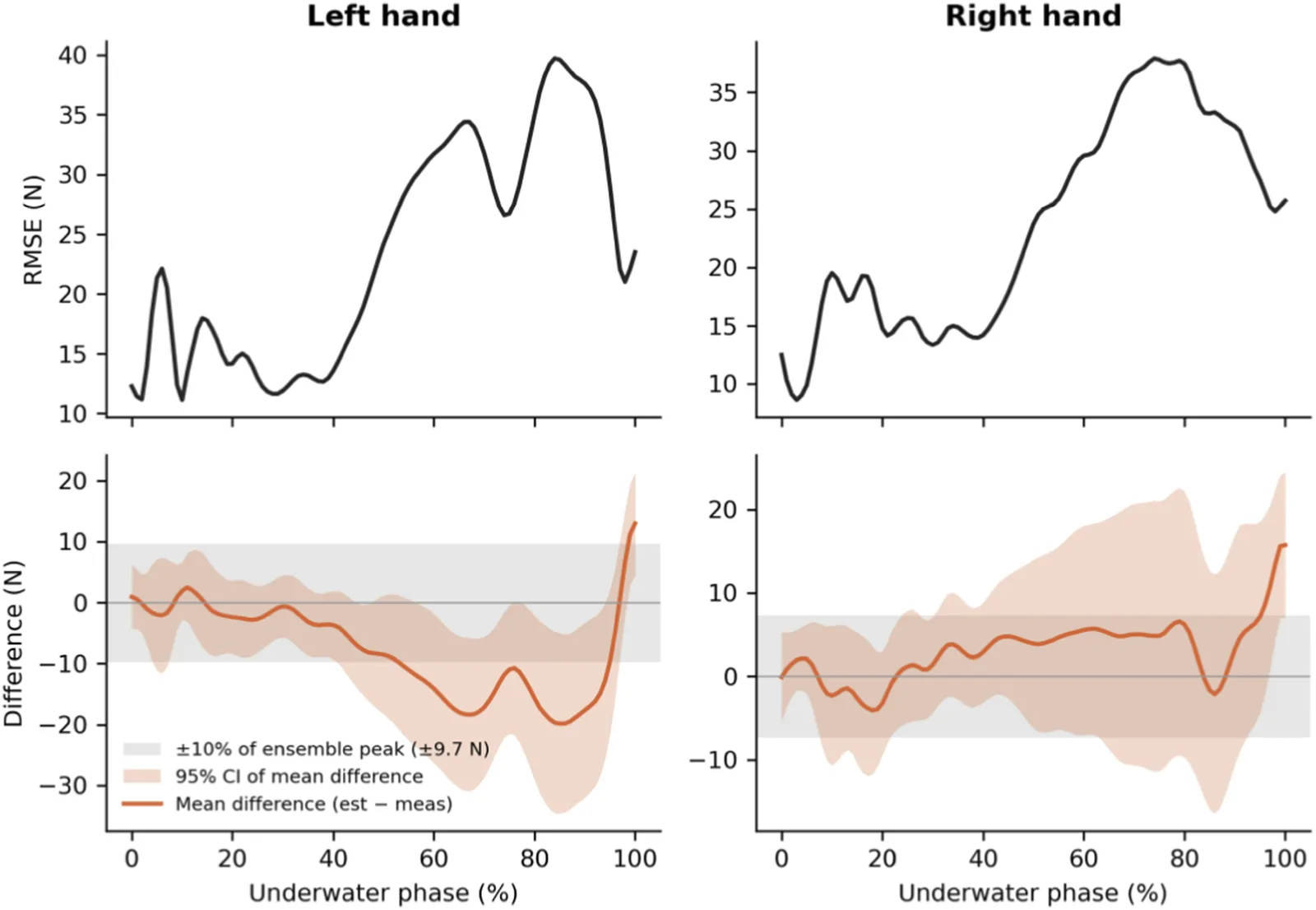
Primary target (resultant force). Top: RMSE across swimmers at each point of the underwater phase. Bottom: mean paired difference (estimated − measured; line) with its pointwise 95% confidence interval (orange band) and the ±10%-of-ensemble-peak equivalence region (grey band). n = 24 per hand. The pointwise interval is not corrected for multiple comparisons; the SPM results are in Table 7.

### 3.4 Discrete variables

Estimated impulse and mean force were lower than measured for the left hand (−5.8 N·s and −7.6 N; both p = 0.002, dz ≈ −0.7) and not detectably different for the right (+2.4 N·s and +3.1 N; p = 0.35). Mean absolute percentage errors were 20% (left) and 39% (right) (Table 11; Figure 7). No force-magnitude variable met the ±10% equivalence criterion (TOST p ≥ 0.38 before correction). The between-swimmer correlation between measured and estimated values ranged from −0.36 to 0.27 for peak force, impulse and mean force, and none differed from zero. Time to peak showed a negligible signed bias (+1.5 and +0.8 percentage points) and passed the ±10-point TOST before but not after Holm correction (p = 0.026 and 0.012; Holm p = 0.185 and 0.092). The absolute timing error, however, averaged 12.4 ± 16.1 and 13.1 ± 13.0 points (median 8.2 and 9.8; maximum 77 and 54). It was within five points in 42% and 38% of swimmers and within 10 points in 67% and 50%. The small signed bias therefore reflects cancellation of larger individual errors in both directions.

**TABLE 11:** Discrete stroke variables of the resultant force: measured versus estimated (n = 24 per hand).

Hand	Variable	Measured	Estimated	Bias	p (test)	dz	r (p)	MAPE (%)	TOST p (Holm)
Left	Peak force (N)	116.0 ± 20.9	83.4 ± 10.5	−32.56	<0.001 (t)	−1.46	0.12 (0.591)	28.2	1.000 (1.000)
Left	Impulse (N·s)	39.9 ± 6.3	34.1 ± 4.7	−5.75	0.002 (t)	−0.72	−0.05 (0.832)	20.4	0.854 (1.000)
Left	Mean force (N)	51.8 ± 10.6	44.1 ± 6.4	−7.63	0.002 (t)	−0.70	0.27 (0.208)	20.4	0.861 (1.000)
Left	Time to peak (%)	69.5 ± 15.9	71.0 ± 7.7	1.50	0.820 (W)	0.07	−0.43 (0.038)	—	0.026 (0.185)
Right	Peak force (N)	90.2 ± 29.9	82.7 ± 16.8	−7.59	0.320 (t)	−0.21	−0.16 (0.458)	36.4	0.425 (1.000)
Right	Impulse (N·s)	31.4 ± 8.8	33.8 ± 6.2	2.42	0.351 (t)	0.19	−0.36 (0.081)	38.7	0.391 (1.000)
Right	Mean force (N)	40.3 ± 12.1	43.4 ± 8.0	3.08	0.351 (t)	0.19	−0.21 (0.328)	38.7	0.385 (1.000)
Right	Time to peak (%)	70.8 ± 16.5	71.5 ± 7.2	0.75	0.649 (W)	0.04	−0.09 (0.684)	—	0.012 (0.092)

t, paired t-test; W, Wilcoxon signed-rank (Shapiro–Wilk p < 0.05 for the differences). r, between-participant correlation of measured and estimated values. TOST, equivalence bounds: ±10% of the measured mean for peak, impulse and mean force; ±10 percentage points of the underwater phase for time to peak. Holm adjustment across the eight TOSTs (exploratory family).

**FIGURE 7:**
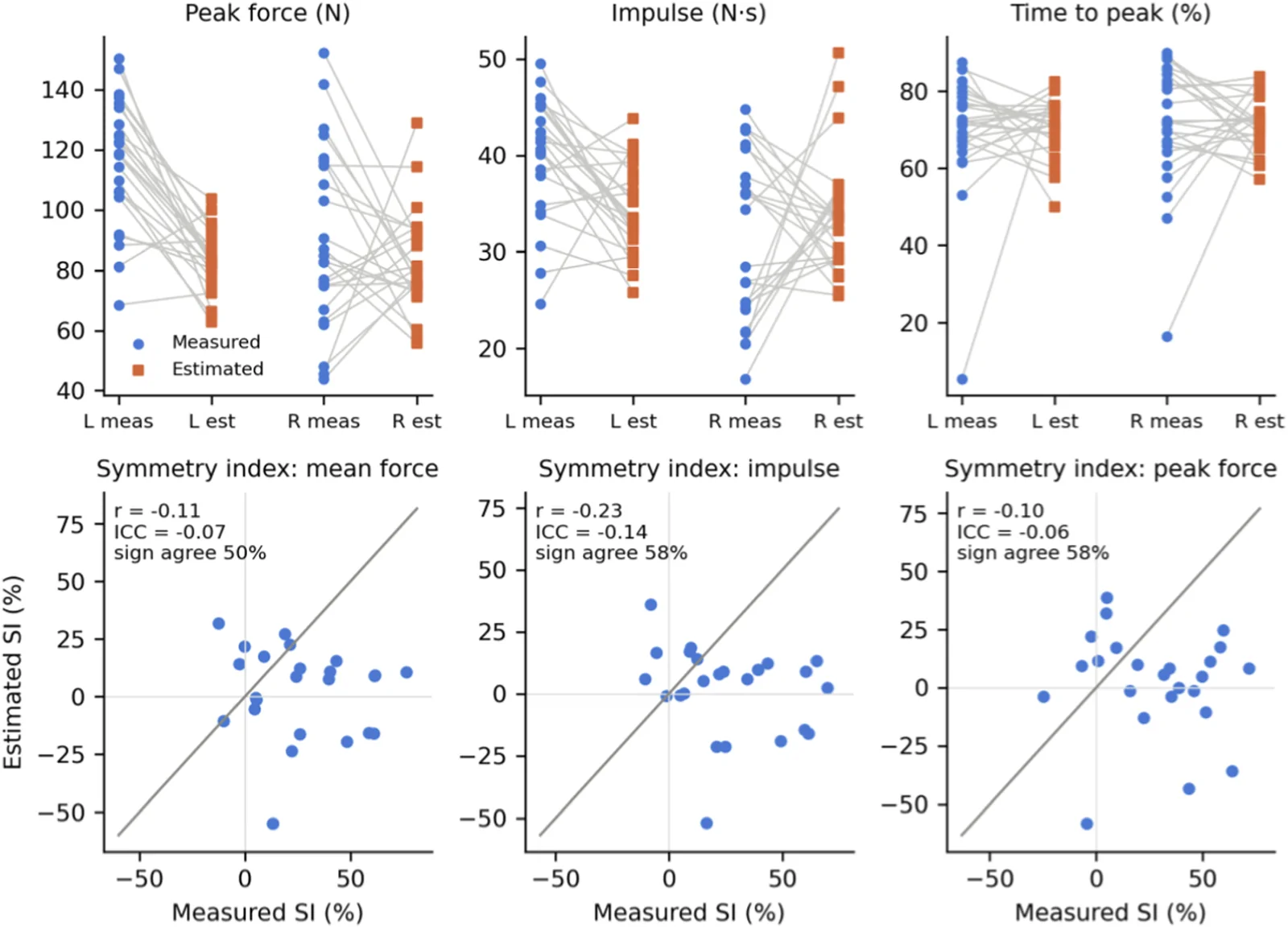
Primary target: participant-level discrete variables and inter-limb symmetry (n = 24). Top: peak force, impulse and time to peak for each swimmer, measured (blue circles) and estimated (orange squares), joined by grey lines. Bottom: estimated versus measured symmetry index, SI = 200 × (L − R)/(L + R), for mean force, impulse and peak force, with the identity line; panel text gives Pearson r, ICC(A,1) and the percentage of swimmers whose dominant side was identified correctly.

Absolute time-to-peak error (percentage points of the underwater phase): left 12.4 ± 16.1 (median 8.2, IQR 3.6–14.0, max 77.0; within five points in 42% and within 10 points in 67% of swimmers); right 13.1 ± 13.0 (median 9.8, IQR 3.6–17.1, max 53.7; within five points in 38% and within 10 points in 50% of swimmers).

### 3.5 Inter-limb symmetry

The measured symmetry index averaged 26.7% ± 25.3% for mean force, 26.0% ± 24.8% for impulse and 28.2% ± 26.4% for peak force (left dominant in 20 of 24 swimmers), whereas the estimated indices averaged 2.3%, 1.6% and 2.1% (Table 12; Figure 7). Agreement was absent. Correlations were −0.11, −0.23 and −0.10, ICC(A,1) values were −0.07, −0.14 and −0.06, and the mean absolute error was 33–38 percentage points. The dominant side was identified correctly in 50%–58% of swimmers, which is chance level.

**TABLE 12:** Agreement of the inter-limb symmetry index, SI = 200 × (L − R)/(L + R) (n = 24).

Variable	Measured SI (%)	Estimated SI (%)	Bias [95% LoA] (points)	Mean absolute error (points)	r (p)	ICC(A,1)	Same dominant side (%)
Mean force	26.7 ± 25.3	2.3 ± 19.7	−24.4 [−90.6, 41.9]	32.8 ± 25.2	−0.11 (0.593)	−0.07	50
Impulse	26.0 ± 24.8	1.6 ± 18.1	−24.4 [−90.8, 42.0]	32.9 ± 25.3	−0.23 (0.277)	−0.14	58
Peak force	28.2 ± 26.4	2.1 ± 22.5	−26.1 [−97.2, 44.9]	37.9 ± 23.0	−0.10 (0.659)	−0.06	58

### 3.6 Variance decomposition, individual records and fold stability

Within-window variation accounted for 92.0% (left) and 85.3% (right) of measured variance, leaving 8.0% and 14.7% between participants (Table 13). The model reproduced the within-window component (mean correlation of mean-removed waveforms 0.814 and 0.808) but not the between-participant amplitude (r = 0.273, p = 0.20; r = −0.202, p = 0.34). Individual records show this pattern (Figure 8). For the best-estimated swimmer, both hands were tracked closely (RMSE 14.3 and 11.0 N; r 0.88 and 0.90). For the median swimmer, timing was reproduced (r 0.89 and 0.79) but amplitude was not (RMSE 25.8 and 22.2 N). For the worst, the estimate stayed near the group-typical waveform while the measured force departed from it (RMSE 29.6 and 42.5 N; r 0.58 and 0.13).

**TABLE 13:** Decomposition of measured resultant-force variance and corresponding agreement of the estimates (n = 24 per hand).

Signal	Within-window variance	Between-participant variance	Within-window r	Amplitude r (p)
Left hand	92.0%	8.0%	0.814	0.273 (0.196)
Right hand	85.3%	14.7%	0.808	−0.202 (0.344)

Within-window r: mean correlation of mean-removed measured and estimated waveforms. Amplitude r: between-participant correlation of measured and estimated window-mean force.

**FIGURE 8:**
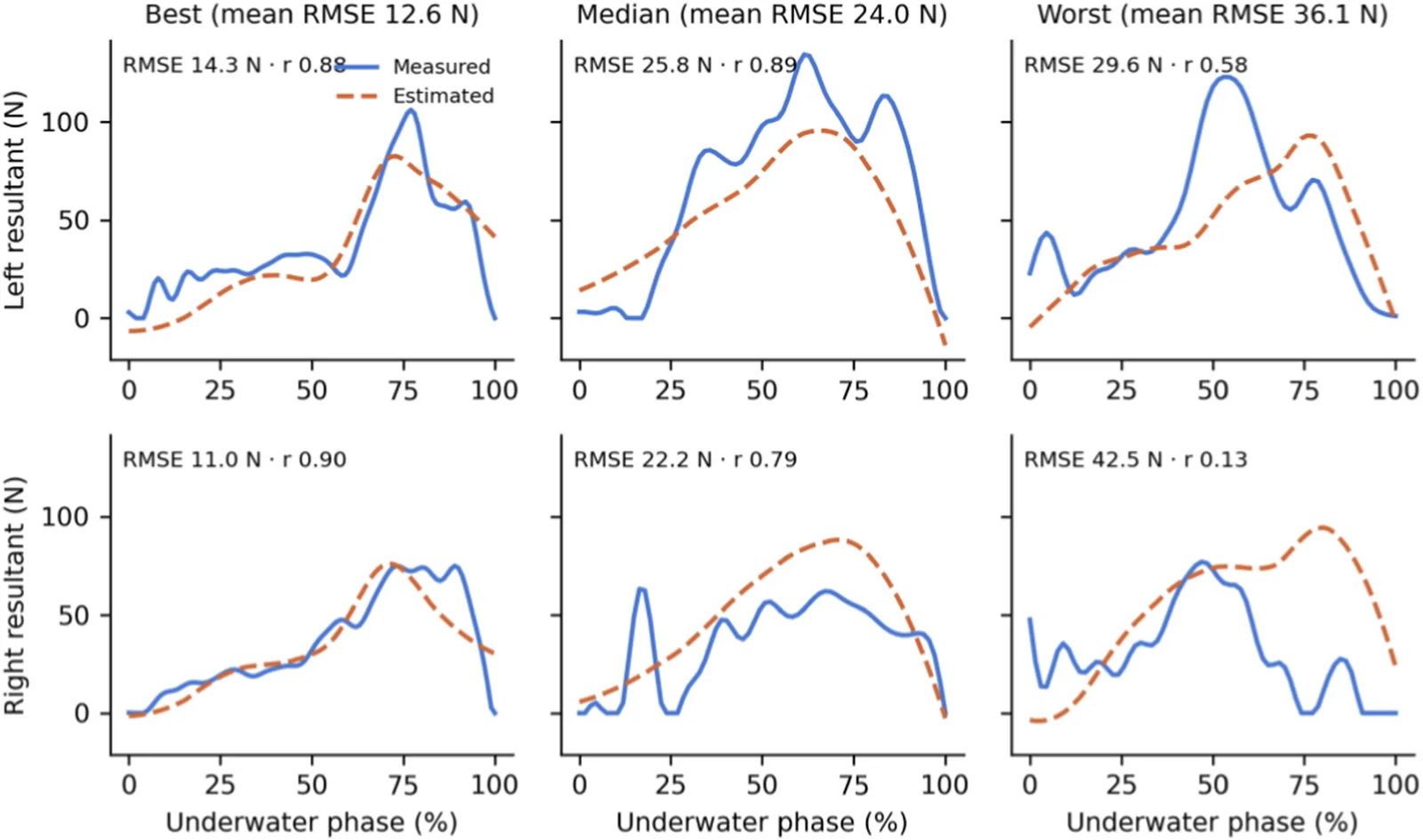
Measured (solid) and estimated (dashed) resultant force over the underwater phase of each hand for the swimmers with the best, median and worst mean RMSE.

Fold-level performance (Table 14) was stable for the left hand (R^2^ 0.41–0.63) and unstable for the right (R^2^ −0.67 to 0.68). In fold 3 the right-hand estimates for the four held-out swimmers were less accurate than predicting the training-fold mean. The selected architectures differed across folds (two-layer or three-layer networks, α 0.01–1.0), which is consistent with a flat selection surface; the smoothing window reached the largest candidate (31 frames) in four folds.

**TABLE 14:** Locked configuration and performance of the proposed network in each outer fold (resultant force; fold-level aggregation, pooled frames of the four held-out swimmers).

Fold	Selected architecture	α	SG window (frames)	Left R^2^	Right R^2^	Left RMSE (N)	Right RMSE (N)
1	197–256–128–1	0.01	21	0.525	0.683	25.4	18.1
2	197–256–128–1	0.01	15	0.408	0.144	25.9	24.1
3	197–128–64–32–1	1.0	31	0.576	−0.668	24.9	37.2
4	197–128–64–32–1	1.0	31	0.633	0.378	21.8	20.9
5	197–256–128–1	1.0	31	0.562	0.389	23.4	22.8
6	197–256–128–1	0.01	31	0.514	0.637	27.1	23.8
Mean ± SD	​	​	​	0.536 ± 0.076	0.260 ± 0.495	24.8 ± 1.9	24.5 ± 6.6

Architecture, α and Savitzky–Golay window were selected inside each outer fold on that fold’s four internal-validation swimmers only; the five-seed ensemble used seeds 0–4. Fold-level RMSE, pools frames and therefore differs slightly from the window-level mean in Tables 5 and 9.

### 3.7 Feature importance, anthropometry and sensitivity analyses

Validation R^2^ was most sensitive to permuting the linear-velocity family (mean loss 0.53, range across folds 0.37–0.66). Every other family had a mean effect of 0.10 or less, with ranges that included zero (marker positions 0.10, range −0.04 to 0.19; accelerations 0.03; orientations 0.02; Table 4). Hand length, wrist breadth and their product were not associated with measured mean force (|r| ≤ 0.24, p ≥ 0.26) or with residual amplitude error (partial |r| ≤ 0.09, p ≥ 0.69); hand dimensions varied little (CV 7%) relative to force (CV 21%–30%).

The two handsets’ event-based offsets agreed within 2 frames in 16 of 24 trials but differed by up to 14 frames (Table 15), which supports hand-specific alignment. With the applied alignment, 2.1% ± 3.9% of measured impulse was recorded while the fingertip was out of the water. The device hand velocity lagged marker-derived fingertip speed by a median of eight frames (IQR 3–15). Re-aligning force by that lag and re-training changed RMSE by about 1 N and did not change any conclusion (Table 15). Excluding windows with interpolated marker frames (7 left, 3 right) likewise left the results unchanged.

**TABLE 15:** Synchronisation checks and sensitivity analyses (resultant force).

Check	Result
Difference between the two handsets’ event-based offsets within a trial (frames, 1 frame = 10 ms)	Median 1 (IQR 0–3), max 14; identical in 9/24 trials, ≤2 frames in 16/24
Share of measured impulse recorded while the fingertip was >30 mm above the water surface	2.1% ± 3.9% (max 20.0%)
Shift minimising that share (frames)	Median 0.5 (IQR −2.0 to 8.2)
Lag of device hand velocity behind marker-derived fingertip speed (frames; cross-correlation r)	Median 8 (IQR 3–15); r median 0.76
Force re-aligned 8 frames earlier and model re-trained, left hand	RMSE 23.5 ± 6.8 N (primary 24.5 ± 6.1); r 0.853; CMC 0.841; absolute time-to-peak error 9.3 points
Force re-aligned 8 frames earlier and model re-trained, right hand	RMSE 22.4 ± 8.1 N (primary 23.3 ± 9.9); r 0.842; CMC 0.785; absolute time-to-peak error 11.7 points
Windows without any interpolated marker frame, left hand (n = 17)	RMSE 23.8 ± 5.7 N; r 0.831; CMC 0.830 (all windows: RMSE 24.5 ± 6.1 N; r 0.849; CMC 0.830)
Windows without any interpolated marker frame, right hand (n = 21)	RMSE 23.5 ± 10.0 N; r 0.850; CMC 0.802 (all windows: RMSE 23.3 ± 9.9 N; r 0.844; CMC 0.793)

Finally, when the pipeline was run under a broader window definition in which both hands were modelled in every window, so that each hand’s samples included portions outside its own underwater phase, apparent accuracy increased (left RMSE 21.6 N, per-window r 0.894, R^2^ 0.673; right 21.6 N, 0.850, 0.459), whereas the phase-conditioned template performed poorly under that definition (Table 16). Accuracy estimates therefore depend strongly on the stroke-phase definition.

## 4 Discussion

The study asked whether upper-limb kinematics can stand in for a hand-force device in sprint front crawl, and at which level of the signal. Within the underwater phase, the network reproduced the group-typical force–time pattern of each hand with moderate agreement (r ≈ 0.85, ICC 0.64–0.70). That pattern was reproduced at least as well by a template that knows nothing about the individual swimmer, and the estimates carried no detectable information about which swimmers produced more force or which limb dominated. The central finding is therefore that the network recovers the shape of a typical stroke, whereas, with the kinematic inputs, models and cohort evaluated here, the individual magnitudes that distinguish swimmers or limbs were not recovered. This does not show that kinematics in general lack that information.

Two design choices strongly influence how accurate such an estimator appears. The first is the analysis window. If both hands are modelled in every window, about one-third (33%) of each hand’s samples lie outside its own underwater phase, largely during recovery and entry, when the device force is near zero and the hand is visibly above or entering the water. Recognising these portions from kinematics is easy and inflates correlation, CMC and R^2^. Under such a broader definition the same pipeline reached markedly higher apparent accuracy (Table 16), whereas restricting each hand to its own underwater phase (Chollet et al., 2000; Santos et al., 2022) reduced it. The second is the benchmark set. A phase-conditioned template is a natural reference for any cyclic-waveform estimator because it isolates how much accuracy comes from knowing the typical shape. Here the template was at least as accurate as every learned model, and relative to the template the network showed no advantage over simpler learned models. We recommend both safeguards for kinematics-to-kinetics studies of cyclic sport movements.

**TABLE 16:** Sensitivity analysis: broader window definition in which both hands were modelled in every window (resultant force).

Analysis	Hand	RMSE (N)	Per-window r	CMC	R^2^
Proposed network, broader window	Left	21.6 ± 6.6	0.894	0.868	0.673
Proposed network, broader window	Right	21.6 ± 9.9	0.850	0.821	0.459
Phase-conditioned template, broader window	Left	37.2 ± 8.7	0.298	0.319	0.055
Phase-conditioned template, broader window	Right	30.6 ± 8.6	0.283	0.327	0.029
Proposed network, underwater-phase window (primary analysis)	Left	24.5 ± 6.1	0.849	0.830	0.540
Proposed network, underwater-phase window (primary analysis)	Right	23.3 ± 9.9	0.844	0.793	0.345

In the broader definition each window is the underwater phase of one hand, but both hands are modelled in every window, so about one-third (33%) of each hand’s samples lie outside its own underwater phase (mainly recovery and entry). Under that definition the phase-conditioned template, which is aligned to the window rather than to each hand’s own phase, is a poor reference.

Several explanations for the failure to recover magnitude are plausible, but the present data do not establish which applies; they should be read as hypotheses. The first is hydrodynamic. Hand force depends on hand velocity, orientation, effective area and unsteady flow (Bixler and Riewald, 2002; Marinho et al., 2011; Takagi et al., 2021). The marker set describes velocity and orientation, which is consistent with validation performance being most sensitive to the velocity family and with timing and shape being recovered, but it does not describe effective area, finger spread or the flow field. Hand length and wrist breadth, which barely varied in this homogeneous cohort, did not explain residual error. The second is statistical. Only 8%–15% of the measured variance lay between swimmers, so the signal that distinguishes individuals was small relative to the common waveform, and regularised models tend to shrink towards the group mean when that signal is weak, which is consistent with the compressed peaks and sub-unity slopes. For symmetry, the mirrored design is a further candidate explanation. A single mapping serves both hands, so a systematic left–right difference can be represented only if it is expressed in the kinematics. The measured asymmetry (≈ 27% in favour of the left hand) is large. It may partly reflect differences between the two independently operating handsets, which the present data cannot separate from true bilateral differences.

These findings bound the claims that can be made. Non-significant SPM and paired tests indicate only that no difference was detected at n = 24, and the limits of agreement (about ±45–50 N against peaks of 90–116 N) are too wide for individual assessment. No force-magnitude variable met the equivalence criterion. Time to peak passed TOST only before multiplicity correction, and its absolute error (median 8%–10% of the underwater phase) exceeds the differences that technique interventions usually target. Inter-limb symmetry was not recovered, and within-swimmer longitudinal change could not be tested because each swimmer contributed one stroke per hand from a single session. All kinematics were recorded with the handsets worn, so performance without the device (the intended use case) is untested. Accordingly, we do not claim applicability to symmetry assessment, longitudinal monitoring or hardware-free deployment. What the approach supports, within the tested protocol, is a group-level description of the typical force–time pattern, for which a phase template performs equally well.

The comparison with other methods should be read in context. The error magnitude (nRMSE 21%–30%) resembles the upper end of terrestrial ground-reaction-force estimation from kinematics (Ozates et al., 2025), but that task involves contact forces constrained by whole-body dynamics, different populations and a different normalisation. Kadi et al. (2022) reported CMC = 0.99 for a pressure-plus-IMU method, but their criterion was itself a pressure-derived force. Their comparison therefore shares the pressure-based derivation, whereas ours relates kinematics to device-derived force, so the two numbers are not a ranking against a common ground truth.

The present work also differs from sensor-based machine-learning studies in aquatic rehabilitation. Amin and Noh (2024) classified upper-limb movements from muscle-activation signals with an LSTM model, a discrete classification problem, whereas the present task is continuous regression of hydrodynamic force. Combining kinematics with muscle-activation and inertial measurements, with explicit attention to the limitations of each modality (Amin et al., 2023; 2025), is a plausible route to recovering the individual information that kinematics lack. Sequence architectures, in which feature redundancy must be managed, may also help recover peaks, but the template benchmark should be retained in any such work.

## 5 Limitations

Twenty-four male sprinters contributed one underwater phase per hand; mirroring pooled the hands but did not increase the number of independent participants. With 24 swimmers, each outer fold selected its configuration on only four internal-validation swimmers and was tested on four others. Model selection was therefore noisy, as the different architectures chosen across folds indicate (Table 14), and fold-level estimates were imprecise (right-hand R^2^ −0.67 to 0.68), so the pooled out-of-fold estimates, not individual folds, should be interpreted. Women, other strokes, breathing, fatigue and submaximal speeds were not studied. The reference device’s force outputs lack published criterion validation, and its native sampling, calibration and force algorithm are not documented. The estimand is therefore SwimBETTER-derived force, not true hydrodynamic force. Synchronisation relied on kinematic entry and exit events, and the lag between device velocity and marker speed (median 80 ms) could not be resolved into a device-processing delay or a timing offset, although re-alignment did not change the conclusions. Missing contralateral markers were interpolated over gaps of up to 47 frames, again without effect on the conclusions. The analysis plan was not preregistered, so all inference is exploratory. Capture volumes, calibration and manual markers are still required.

## 6 Future directions

Recovering individual magnitude will likely require inputs that encode effective hand area and orientation relative to the flow (for example, hand-shape scans or markerless hand models), a small number of instrumented calibration strokes per swimmer, or multimodal sensing. Any claim of symmetry assessment, longitudinal monitoring or device-off deployment will require direct validation: agreement of asymmetry indices, repeated sessions with known changes, and kinematics recorded without the device. Evaluations should report a phase-conditioned template and restrict windows to the underwater phase.

## 7 Conclusion

Within the underwater phase and a device-on protocol, a kinematics-driven artificial neural network reproduced the typical force–time pattern of each hand in sprint front crawl with moderate agreement. It was no more accurate than a phase-conditioned template and did not recover between-swimmer force magnitude or inter-limb asymmetry. With the inputs and models evaluated here, kinematics-only estimation is therefore not yet a substitute for hand-force measurement for individual swimmers.

## Data Availability

The raw data supporting the conclusions of this article will be made available by the authors, without undue reservation.
